# 4-Methyl-3-penten-2-one – Determination of 4-methyl-3-penten-2-one (mesityl oxide) in the workplace air using high-performance liquid chromatography (HPLC-DAD): Air Monitoring Method – Translation of the German version from 2025

**DOI:** 10.34865/am14179e10_4or

**Published:** 2025-12-22

**Authors:** Lutz Nitschke, Adela Frenzen, Dietmar Breuer, Ralph Hebisch, Uta Lewin-Kretzschmar, Andrea Hartwig, MAK Commission

**Affiliations:** 1 Bavarian State Office for Health and Food Safety (LGL) Pfarrstraße 3 80538 München Germany; 2 Hochschule Bonn-Rhein-Sieg. Department of Natural Sciences Grantham-Allee 20 53757 Sankt Augustin Germany; 3 Federal Institute for Occupational Safety and Health (BAuA) Friedrich-Henkel-Weg 1–25 44139 Dortmund Germany; 4 German Social Accident Insurance, Institution for the raw materials and chemical industry, Prevention - Department of Hazardous Substances, Biological Agents and Analytical Chemistry Kurfürsten-Anlage 62 69115 Heidelberg Germany; 5 Institute of Applied Biosciences. Department of Food Chemistry and Toxicology. Karlsruhe Institute of Technology (KIT) Adenauerring 20a, Building 50.41 76131 Karlsruhe Germany; 6 Permanent Senate Commission for the Investigation of Health Hazards of Chemical Compounds in the Work Area. Deutsche Forschungsgemeinschaft, Kennedyallee 40, 53175 Bonn, Germany. Further information: Permanent Senate Commission for the Investigation of Health Hazards of Chemical Compounds in the Work Area | DFG

**Keywords:** 4-methyl-3-penten-2-one, air analyses, analytical method, workplace measurement, hazardous substance, high-performance liquid chromatography, diode array detection, HPLC-DAD, silica gel, liquid desorption, Luft, air

## Abstract

The working group “Air Analyses” of the German Senate Commission for the Investigation of Health Hazards of Chemical Compounds in the Work Area (MAK Commission) developed and verified the presented analytical method. It is used to determine the levels of 4-methyl-3-penten-2-one [141-79-7] that occur in the workplace air. The method covers concentrations in the range from one tenth up to twice the current occupational exposure limit value (OELV) of 8.1 mg/m^3^. The method is also suitable for verifying the short-term exposure limit (STEL; excursion factor 2). Samples are collected by drawing a defined volume of air through a sampling tube filled with silica gel using a flow regulated pump at a volumetric flow rate of 0.5 l/min. Exposure during the shift is measured with a sampling period of 2 hours and the short-term exposure with a period of 15 minutes. The 4-methyl-3-penten-2-one adsorbed to the silica gel is extracted by liquid extraction with methanol and analysed by high-performance liquid chromatography using diode array detection. The quantitative determination is based on multiple-point calibrations with external standards. A relative limit of quantification (LOQ) of 0.06 mg/m^3^ is obtained for an air sample volume of 60 litres. As the LOQ for a sample volume of 30 litres is 0.03 mg/m^3^, the STEL can also be measured. The recovery is approx. 100% and the expanded uncertainty is 14% for a sampling period of 2 hours and below 16% for a period of 15 minutes.

**Table:** 

**Method number**	1
**Application**	Air analysis
**Analytical principle**	High-performance liquid chromatography with diode array detection (HPLC-DAD)

## 1 Characteristics of the method

**Table:** 

**Precision:**	Standard deviation (rel.):	*s* = 1.1–1.4%
	Expanded uncertainty:	*U* = 14%
	in the concentration range of 0.81–16.2 mg/m^3^ and n = 6 determinations	
**Limit of quantification:**	3.5 µg absolute per sample carrier	
	0.06 mg/m^3^ for an air sample volume of 60 l and a sampling period of 2 h	
**Recovery:**	*η* = 100.4–101.4%	
**Sampling recommendations:**	Sampling period:	2 h
	Air sample volume:	60 l
	Volumetric flow rate:	0.5 l/min
	For short-term measurements:	15 min; 2 l/min

## 2 Description of the substance

### 4-Methyl-3-penten-2-one [141-79-7]

**Fig. 1:**
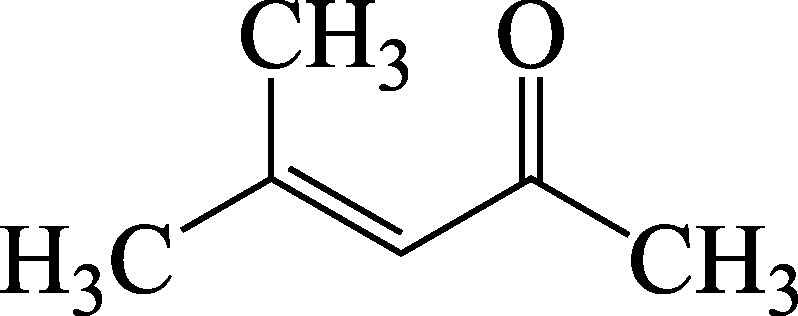
Structural formula of 4-methyl-3-penten-2-one

4-Methyl-3-penten-2-one (see Figure 1, also known as mesityl oxide, isopropylidene acetone, 1-isobutenyl methyl ketone, methyl isobutenyl ketone) is a colourless to yellowish oily liquid with an odour reminiscent of peppermint. In industry, 4-methyl-3-penten-2-one is produced by the dehydration of diacetone alcohol, which is a product of the aldol reaction of acetone (IFA 2024; RÖMPP-Redaktion 2002). Technical-grade 4-methyl-3-penten-2-one contains up to 10% of the isomer 4-methyl-4-penten-2-one [3744-02-3].

Most of the 4-methyl-3-penten-2-one is further processed to 4-methylpentan-2-one [108-10-1] (methyl isobutyl ketone, MIBK), which is used in the lacquer industry as a solvent for natural and synthetic resins. 4-Methyl-3-penten-2-one also serves as a precursor in the synthesis of specific terpenes and odorants (RÖMPP-Redaktion 2002; RÖMPP-Redaktion and Jahn 2019).

The occupational exposure limit value (OELV) and the MAK value have both been set at 8.1 mg/m^3^ (2 ml/m^3^) for the workplace air. The substance has been classified in Peak Limitation Category I with an excursion factor of 2 based on its short-term concentration. The substance has been designated with an “H” (for substances which can be absorbed through the skin in toxicologically relevant amounts) (AGS 2024; DFG 2024). The substance data for 4-methyl-3-penten-2-one are given in Table 1.

**Tab. 1:** Substance data for 4-methyl-3-penten-2-one (IFA 2024)

Name	4-methyl-3-penten-2-one
CAS No.	141-79-7
Molar mass [g/mol]	98.14
Physical state at 20 °C	liquid
Density at 25 °C [g/cm^3^]	0.858^a)^
Vapour pressure at 20 °C [hPa]	10.9
Melting point [°C]	–59
Boiling point at 1013 hPa [°C]	130
Flash point [°C]	24
Criteria of assessment	
Germany: OELV, MAK value (AGS 2024; DFG 2024)	8.1 mg/m^3^
Peak limitation category (excursion factor) (AGS 2024; DFG 2024)	I(2)

^a)^ density as specified on the safety data sheet of the manufacturer (Sigma-Aldrich 2019)

## 3 General principles

This analytical method is used to determine the 4-methyl-3-penten-2-one concentration in the workplace air in the range of one tenth to twice the currently valid OELV or MAK value of 8.1 mg/m^3^ (AGS 2024; DFG 2024). The method is also suitable for monitoring compliance with the short-term concentration with an excursion factor of 2 (DIN 2021 a). The method complies with the technical rules for hazardous substances TRGS 402 (AGS 2023) in all respects.

Samples are taken by drawing a defined volume of air through a sorbent tube (silica gel) using a suitable pump. The contents of the loaded silica gel tube are transferred to an amber glass bottle, covered with a layer of methanol, and shaken. The sample is analysed in the methanol extract by high-performance liquid chromatography (HPLC) with diode array detection (DAD). The quantitative evaluation is carried out with external standards using a calibration curve.

## 4 Equipment, chemicals and solutions

### 4.1 Equipment

For sampling:

- Sampling pump for personal or stationary sampling, suitable for a volumetric flow rate of 0.5 l/min (e.g. SG5200, from GSA Messgerätebau GmbH, 40880 Ratingen, Germany)
- Silica gel tubes, activated silica gel (45/60) 300/150 mg, 8 × 75 mm (e.g. ORBO 506, Supelco, from Merck KGaA, 64271 Darmstadt, Germany); at higher humidity levels: higher capacity silica gel tubes, type B/G, purified silica gel, 1100/480 mg, 7 × 122 mm (e.g. type B/G, from Drägerwerk AG & Co. KGaA, 23558 Lübeck, Germany)
- Flow meter (e.g. Bios DryCal Definer 220, from MesaLabs, supplied by Brandt Instruments, Inc., Prairieville, LA, USA)
- Silicone tubing of suitable dimensions to connect the sampling pump and silica tubes
- Hygrometer to measure humidity

For sample preparation and the analytical determination:

- Ultrapure water system for the preparation of ultrapure water (e.g. Millipore-Q-Integral 3, from Merck KGaA, 64271 Darmstadt, Germany)
- Variable positive-displacement piston pipettes, 10–100 µl and 100–1000 µl (e.g. from Gilson, Inc., Middleton, WI, USA)
- Pipette tips (e.g. from Gilson, Inc., Middleton, WI, USA)
- Bottle-top dispenser, 10–50 ml (e.g. RotiLabo-Dispenser, from Carl Roth GmbH + Co. KG, 76185 Karlsruhe, Germany)
- Amber glass bottles, about 22 ml, with screw caps (e.g. Supelco, from Merck KGaA, 64271 Darmstadt, Germany)
- Laboratory shaker (e.g. IKA-VIBRAX-VBA, from Jahnke & Kunkel GmbH & Co. KG, 79219 Staufen, Germany)
- Volumetric flasks made of glass, 10 ml, with glass stoppers (e.g. from BRAND GmbH + Co KG, 97877 Wertheim, Germany)
- Disposable syringes, 10 ml, with Luer-Lock connection (e.g. from Henke-Sass, Wolf GmbH, 78532 Tuttlingen, Germany)
- Syringe filters, regenerated cellulose, pore size 0.45 µm, Ø 25 mm, with Luer-Lock connection (e.g. Chromafil RC-45/25, from Macherey-Nagel GmbH & Co. KG, 52355 Düren, Germany)
- High-performance liquid chromatograph, autosampler with cooling unit, column oven and DAD (e.g. Dionex UltiMate 3000, from ThermoFisher Scientific GmbH, 63303 Dreieich, Germany)
- Autosampler vials, 1.5 ml, 11.6 × 32 mm, amber glass, threaded, with cap (e.g. from Macherey-Nagel GmbH & Co. KG, 52355 Düren, Germany)
- Separation column, C18, length (L) 250 mm; inner diameter (ID) 4.6 mm; particle size: 5 μm with pre-column L 20 mm, ID 4 mm, particle size 5 μm (e.g. Supelcosil LC‑18 with Supelguard LC-18, from Merck KGaA, 64271 Darmstadt, Germany)
- Tube opener (e.g. TO 7000, from Drägerwerk AG & Co. KGaA, 23558 Lübeck, Germany)

### 4.2 Chemicals

- 4-Methyl-3-penten-2-one, certified reference material (e.g. PHR1547, Sigma-Aldrich, from Merck KGaA, 64271 Darmstadt, Germany)
- Methanol, HPLC-grade, ≥ 99.9% (e.g. Merck KGaA, 64271 Darmstadt, Germany)
- Acetonitrile, HPLC-grade, ≥ 99.95% (e.g. Rotisolv HPLC Ultra Gradient Grade, from Carl Roth GmbH + Co. KG, 76185 Karlsruhe, Germany)
- Ultrapure water (ρ ≥ 18.2 MΩ × cm at 25 °C)

### 4.3 Solutions

The following solutions are prepared using the chemicals listed in Section 4.2. The solutions can be stored for at least 3 months in the refrigerator at 4 to 8 °C.

**Stock solution**: (48.6 mg/ml in methanol)

486 mg of 4-methyl-3-penten-2-one reference material (566 µl, ρ = 0.858 g/cm^3^) is transferred to a 10-ml volumetric flask containing about 2 ml of methanol. The flask is filled to the mark with methanol and then shaken.

The working solutions are prepared by diluting the stock solution.

**Working solution 1 (spiking solution 1)**: 1:5 dilution of the stock solution (9.72 mg/ml in methanol)

About 2 ml of methanol is placed into a 10-ml volumetric flask. 2 ml of the stock solution is added, the flask is filled to 10 ml with methanol and then shaken.

**Working solution 2 (spiking solution 2)**: 1:10 dilution of the stock solution (4.86 mg/ml in methanol)

About 2 ml of methanol is placed into a 10-ml volumetric flask. 1 ml of the stock solution is added, the flask is filled to 10 ml with methanol and then shaken.

**Working solution 3 (spiking solution 3)**: 1:100 dilution of the stock solution (486 µg/ml in methanol)

About 2 ml of methanol is placed into a 10-ml volumetric flask. 0.1 ml of the stock solution is added, the flask is filled to 10 ml with methanol and then shaken.

**Calibration solutions**: (calibration solution 1: 97.2 µg/ml, calibration solution 2: 48.6 µg/ml, calibration solution 3: 4.81 µg/ml in methanol)

The calibration solutions 1, 2 and 3 are prepared from the working solutions 1, 2 and 3 using 1:100 dilutions with methanol. 0.1 ml of the respective working solution is transferred to a 10-ml volumetric flask containing about 2 ml of methanol, the flasks are then filled to the mark with methanol and shaken.

### 4.4 Calibration standards

The calibration standards are prepared by diluting the calibration solutions with methanol according to the pipetting scheme given in Table 2. Piston pipettes are used to transfer the calibration solutions and the solvent (methanol) to 1.5-ml vials in the volumes listed in the table.

**Tab. 2:** Pipetting scheme for preparing the calibration standards and the resulting concentrations

**Calibration standard**	**Calibration solution**	**Concentration of calibration solution** **[µg/ml]**	**Volume of calibration solution** **[µl]**	**Volume of methanol** **[µl]**	**Concentration of calibration standard** **[µg/ml]**	**Mass per 10 µl injection** **[ng]**
I	3	4.86	5	995	0.0243	0.243
II	3	4.86	10	990	0.0486	0.486
III	3	4.86	20	980	0.0972	0.972
IV	3	4.86	25	975	0.122	1.22
V	3	4.86	50	950	0.243	2.43
VI	3	4.86	100	900	0.486	4.86
VII	3	4.86	200	800	0.972	9.72
VIII	3	4.86	400	600	1.94	19.4
IX	3	4.86	1000	0	4.86	48.6
X	1	97.2	100	900	9.72	97.2
XI	2	48.6	333	667	14.6	146
XII	1	97.2	200	800	19.4	194
XIII	2	48.6	500	500	24.3	243
XIV	2	48.6	600	400	29.2	292
XV	2	48.6	700	300	34.0	340
XVI	2	48.6	800	200	38.9	389
XVII	2	48.6	900	100	43.7	437
XVIII	2	48.6	1000	0	48.6	486
XIX	1	97.2	667	333	64.8	648
XX	1	97.2	833	167	81.0	810
XXI	1	97.2	1000	0	97.2	972
XXII	spiking sol. 2	4860	25	975	122	1220

### 4.5 Control solutions

The control solutions that serve as the quality control are prepared with dilutions (1:100) of the working solutions 1, 2 and 3. A dispenser is used to transfer 10 ml of methanol to amber glass bottles. 100 µl of the methanol is then removed by pipette and discarded. 100 µl of working solution 1, 2 or 3 is added to the amber glass bottles, one solution per bottle. The control solution is prepared fresh every working day and analysed under the conditions listed in Section 6.

## 5 Sampling and sample preparation

### 5.1 Sampling

Samples are collected using stationary or personal sampling procedures. The samples taken by personal sampling are collected within the breathing zone. The silica gel tubes are cut open with a glass cutter directly before sampling begins. The sampling tubes are connected to a pump by a length of tubing.

The shift average is determined by drawing an air sample through a silica gel tube for at least 2 hours using a flow-regulated pump operating at a volumetric flow rate of 0.5 l/min. Over a sampling period of 2 hours, this is equivalent to an air sample volume of 60 l. To determine the short-term concentration, the air sample is drawn through the silica gel tube for a period of 15 minutes at a volumetric flow rate of 2 l/min (30 l sampling volume).

A sampling record is kept of the parameters required for determining the concentration in air (air sample volume, temperature and relative humidity). It is important to consider the relative humidity when choosing a suitable sampling system (see Section 10.2).

After sampling, the flow rate must be checked for constancy. If the value deviates from the adjusted flow rate by more than ± 5%, the sample should be taken again (DIN 2023).

### 5.2 Sample preparation

The assessment of the storage stability (see Section 10.6) demonstrated that the silica gel tubes must be prepared within 2 weeks of sample collection. Amber glass bottles are filled with 10 ml of methanol. The entire contents of each silica gel tube (i.e. including the control layer and the glass wool) are transferred to the bottles, one tube per bottle. The amber glass bottles are placed into a laboratory shaker and shaken for 30 minutes at 400 to 500 revolutions per minute. The extracts are then filtered and aliquots are transferred to 1.5-ml autosampler vials. The vials are then placed into an autosampler and analysed by HPLC.

The lab blank is determined by spiking one silica gel tube of the batch being used with 100 µl of methanol. The lab blank is prepared and analysed in the same manner as the samples.

## 6 Operating conditions

**Table:** 

**Apparatus:**	High-performance liquid chromatograph with autosampler, column oven and DAD, e.g. Dionex Ultimate 3000, ThermoFisher Scientific GmbH
**Pre-column:**	Supelguard LC-18, ID 4 mm, L 20 mm, particle size 5 µm
**Separation column:**	Supelcosil LC‑18, ID 4.6 mm, L 250 mm, particle size 5 µm
**Column temperature:**	30 °C
**Detector:**	Diode array detector
**Wavelength:**	Scan: 200–550 nm, quantification: 239 nm
**Mobile phase:**	Acetonitrile/ultrapure water, 1:1 (v/v), isocratic
**Flow rate:**	0.6 ml/min
**Injection volume:**	10 µl
**Run time:**	12 min

4-Methyl-3-penten-2-one has a retention time of about 8.5 minutes under the specified conditions.

## 7 Analytical determination

After the samples are prepared as described in Section 5.2, the analytical determination is performed by injecting 10 µl of each sample into the high-performance liquid chromatograph. The samples are analysed under the conditions specified in Section 6. If the concentrations obtained are above the calibrated range, suitable dilutions in methanol must be prepared and the analysis carried out again.

## 8 Calibration

**Fig. 2:**
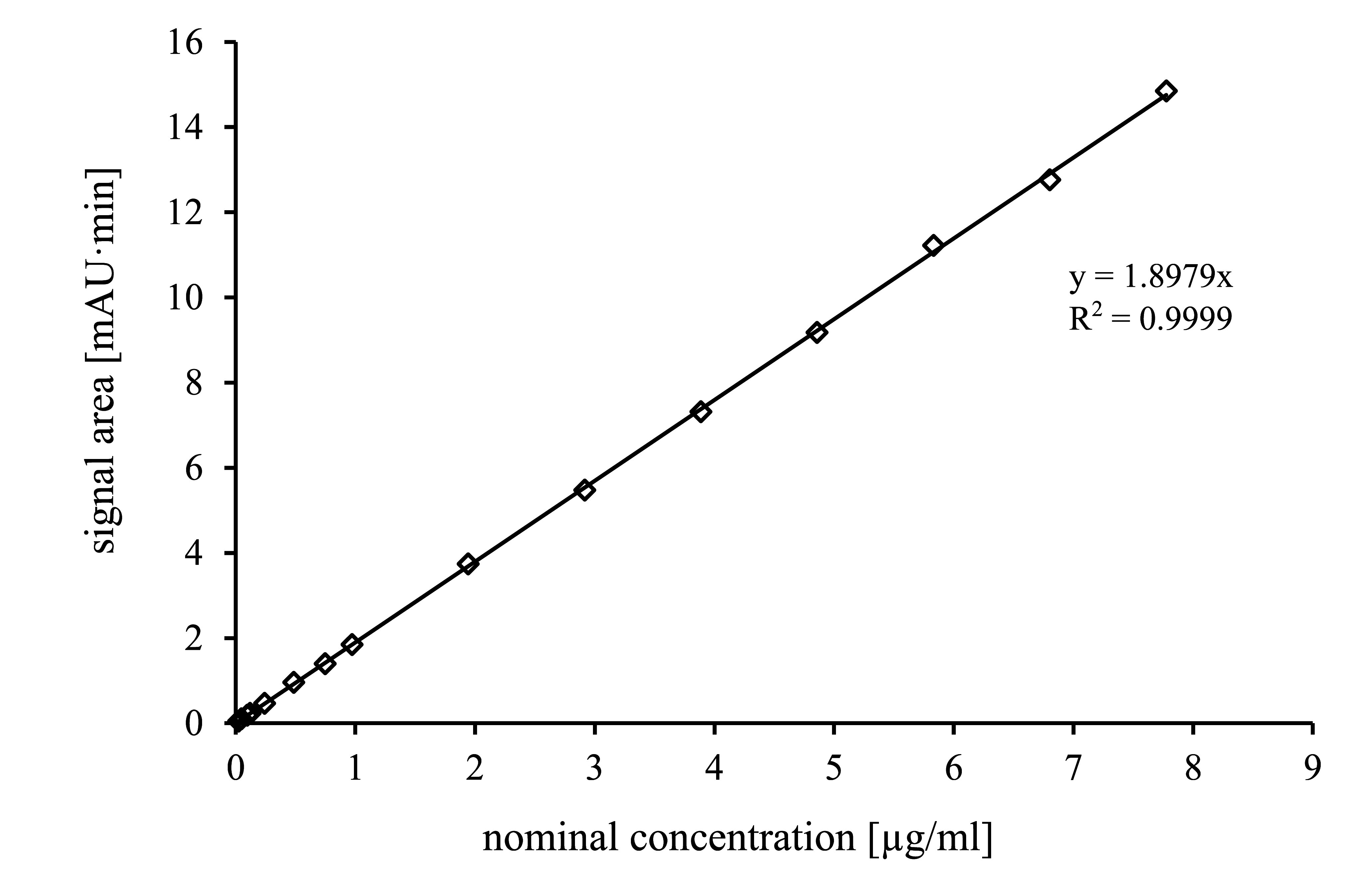
Calibration function of 4-methyl-3-penten-2-one in the concentration range of the LOQ

The calibration function is obtained by analysing the calibration standards described in Section 4.4 according to Sections 6 and 7. The resulting peak areas are plotted against the respective concentrations. The calibration function is linear in the investigated concentration range (see Figure 2).

The calibration function must be verified every working day by analysing quality control solutions (Section 4.5). A re-calibration must be performed if the analytical conditions change or the results of the quality control indicate that this is necessary.

## 9 Calculation of the analytical result

The 4-methyl-3-penten-2-one concentration in the workplace air (*ρ*) is calculated using the concentration in the measurement solution that was determined by the data analysis programme. The data analysis programme performs this calculation based on the determined calibration function. The concentration of 4-methyl-3-penten-2-one in the workplace air is calculated from the concentration in the measurement solution by applying Equation 1 and taking into consideration the desorption volume, the blank value for 4-methyl-3-penten-2-one and the air sample volume.



(1)

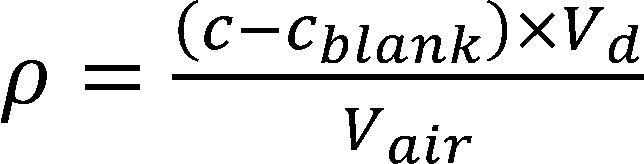

rho equal to startFraction open parenthesis c minus c subscript blank close parenthesis times V subscript d divid-ed by V subscript air endFraction.



Equation 2 is applied to extrapolate the value to 20 °C and 1013 hPa.



(2)

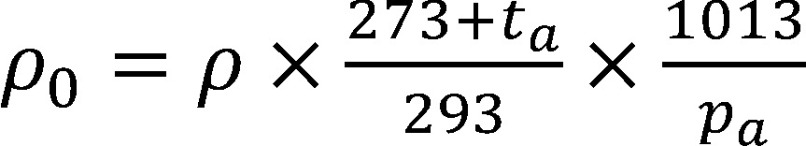

rho subscript 0 equal to rho times startFraction 273 plus t subscript a divided by 293 endFraction times 1013 divided by p subscript a.



where:

**Table:** 

*ρ*	is the mass concentration of 4-methyl-3-penten-2-one in the air sample in mg/m^3^ at *t_a_* and *p_a_*
*ρ_0_*	is the mass concentration of 4-methyl-3-penten-2-one in mg/m^3^ at 20 °C and 1013 hPa
*c*	is the concentration of 4-methyl-3-penten-2-one in the measurement solution in mg/l
*c_blank_*	is the concentration of the blank value in mg/l
*V_d_*	is the volume of the desorption solution in l
*V_air_*	is the air sample volume in m^3^ (determined from the volumetric flow rate and the sampling period, in this case 0.06 m^3^)
*t_a_*	is the temperature during sampling in °C
*p_a_*	is the air pressure during sampling in hPa

## 10 Reliability of the method

The characteristics of the method were determined according to DIN EN 482 (DIN 2021 a), DIN EN ISO 22065 (DIN 2021 b) and DIN 32645 (DIN 2008). The method was fully validated.

### 10.1 Precision, recovery and expanded uncertainty

The precision and expanded uncertainty were determined by spiking sets of 6 silica gel tubes with 4-methyl-3-penten-2-one; three different masses were used. The masses used for spiking were chosen to be equivalent to one tenth of the OELV, the OELV and twice the OELV at an air sample volume of 60 l. For this purpose, sets of 6 silica gel tubes were spiked with 100 µl of spiking solution 1, 2 or 3 (see Section 4.3). Air was then drawn through the spiked silica gel tubes for 2 hours at a temperature of about 25 °C and a relative humidity of about 30% using a pump operating at a flow rate of 0.5 l/min. The sampling tubes were then prepared and analysed according to the steps described in Sections 5.2, 6 and 7. The results were used to calculate the data for precision and recovery given in Table 3.

**Tab. 3:** Recovery, relative standard deviation and expanded uncertainty *U* for n = 6 determinations

**Spiked mass** **[µg]**	**Concentration^a)^** **[mg/m^3^]**	**Recovery** **[%]**	**Standard deviation (rel.)** **[%]**	**Expanded uncertainty *U*** **[%]**
48.6	0.81	100.4	1.4	14.2
486	8.1	101.4	1.1	14.3
972	16.2	101.1	1.4	14.3

^a)^ The concentration is calculated based on a 2-hour sampling period and a flow rate of 0.5 l/min.

The expanded uncertainty was determined by estimating all relevant influencing parameters. The two main sources of uncertainty in the measurement results are uncertainties in the sampling procedure according to Annex B of DIN EN 482 (DIN 2021 a) and uncertainties in the analytical procedure.

A blank value was concurrently determined for each set of spiked samples (see Section 5.2).

### 10.2 Influence of humidity

The influence of humidity on recovery was investigated at room temperature (about 20–25 °C) using concentrations equivalent to 0.1 and 2 times the OELV and a volumetric flow rate of 0.5 l/min. For each analysis, sets of 4 silica gel tubes were spiked with one of the spiking solutions 1, 2 or 3. Air was then drawn through the tubes over the specified sampling period at varying levels of relative humidity using a pump operating at a volumetric flow rate of 0.5 l/min. The results of the experiments using different levels of relative humidity, sampling periods and loads are given in Table 4.

**Tab. 4:** Influence of relative humidity on the recovery of 4-methyl-3-penten-2-one at room temperature

**Rel. humidity** **[%]**	**Spiked mass** **[µg]**	**Sampling period** **[min]**	**Air sample volume** **[l]**	**OELV**	**Recovery** **[%]**	**Standard deviation (rel.)** **[%]**
Silica gel tubes ORBO 506						
20	48.6	120	60	0.1	102.9	0.8
20	972	120	60	2.0	102.0	0.5
30	48.6	120	60	0.1	101.0	0.9
30	972	120	60	2.0	100.3	1.5
50	486	60	30	2.0	98.0	1.2
50	972	120	60	2.0	96.5	1.2
60	24.4	60	30	0.1	101.5	7.6
60	486	60	30	2.0	100.9	4.2
60	48.6	120	60	0.1	63.3	1.4
60	972	120	60	2.0	40.1	11.5
Silica gel tubes Dräger type B/G						
80	24.4	60	30	0.1	97.4	1.6
80	486	60	30	2.0	101.8	1.5
80	48.6	120	60	0.1	95.6	1.6
80	972	120	60	2.0	94.3	2.9

At a relative humidity of 60% and above, the adsorbed 4-methyl-3-penten-2-one is displaced by the flow of the air sample. If this occurs, the sampling period and/or the flow rate must be reduced in order to reduce the sampling volume; the volume of air should not exceed 30 l.

As an alternative, two silica gel tubes (type ORBO 506) are set up in series or a higher-capacity silica gel tube (e.g. Dräger type B/G) is used. The data for the recovery of 4-methyl-3-penten-2-one using Dräger type B/G sampling tubes at a relative humidity of 80% are given in Table 4. The recovery experiments demonstrated that the sampling volume should not be greater than 30 litres at a relative humidity of 80% even if Dräger type B/G silica gel tubes are used.

The results show that the humidity levels must always be determined while sampling!

### 10.3 Influence of temperature

The influence of temperature on recovery was evaluated at 5 °C and 40 °C. The experiments were carried out at a relative humidity of about 20–25%. Two silica gel tubes, each spiked with 100 µl of spiking solution 1 (9.72 mg/ml, see Section 4.2), were prepared to simulate concentrations equivalent to twice the OELV. Air was drawn through the tubes for 2 hours using a pump operating at a volumetric flow rate of 0.5 l/min. The results of the experiments are given in Table 5.

**Tab. 5:** Influence of temperature on the recovery of 4-methyl-3-penten-2-one at a relative humidity of 20–25%

**Temperature** **[°C]**	**Spiked mass** **[µg]**	**Sampling period** **[min]**	**Air volume** **[l]**	**OELV**	**Recovery** **[%]**
5	972	120	60	2.0	100.1
40	972	120	60	2.0	102.8

As shown in Table 5, the ambient air temperature does not have any effect on recovery under the specified conditions.

At temperatures up to 40 °C and a sampling period of 1 hour, the results are not negatively affected by a relative humidity of up to 40% (cf. Section 10.2).

### 10.4 Limit of quantification

The limit of quantification was determined according to the standard DIN 32645 (DIN 2008). After performing a 15-point calibration in the lower concentration range of 0.0243 to 7.78 µg/ml, the limit of quantification was calculated based on an injection volume of 10 µl. The absolute limit of quantification was 3.5 µg 4-methyl-3-penten-2-one or 0.06 mg/m^3^ at an air sample volume of 60 litres (pump flow rate 0.5 l/min and a sampling period of 2 h).

### 10.5 Capacity of the sampling system

The breakthrough behaviour was determined by connecting sets of two silica gel tubes with a length of silicone tubing. The front tube was loaded either with 100 µl of spiking solution 1 to assess concentrations equivalent to twice the OELV or with 200 µl of spiking solution 1 to assess concentrations equivalent to four times the OELV. Air was then drawn through the tubes at room temperature and a relative humidity of about 30% using a sampling pump operating at a volumetric flow rate of 0.5 l/min. There was no evidence of breakthrough after 2 hours of simulated sampling. The recovery was about 102.9%.

### 10.6 Storage stability

The storage stability of the spiked silica gel tubes was determined after storing the tubes at room temperature for periods of 3, 7 and 14 days. Table 6 gives the data for recovery as the mean of the values obtained from duplicate determinations.

**Tab. 6:** Influence of the length of storage of the spiked silica gel tubes on the recovery of 4-methyl-3-penten-2-one at room temperature

**OELV**	**Recovery** **3 days** **[%]**	**Recovery** **7 days** **[%]**	**Recovery** **14 days** **[%]**
0.1	104.8	102.5	99.2
2.0	101.3	98.2	96.1

Table 6 shows that the loaded tubes remain stable over a storage period of 2 weeks.

The storage stability of the extracts was investigated using 6 independent sample solutions. The methanolic sample extracts remained stable for 1 month if stored in the refrigerator (at about 6–8 °C) (see Table 7).

**Tab. 7:** Influence of the length of storage (at about 6–8 °C) of the extraction solutions on recovery

**OELV**	**Recovery** **10 days** **[%]**	**Standard deviation (rel.)** **10 days** **[%]**	**Recovery** **1 month** **[%]**	**Standard deviation (rel.)** **1 month** **[%]**
0.1	101.1	1.0	102.1	1.5
1.0	101.6	0.5	101.9	0.7
2.0	101.7	0.9	100.3	0.8

### 10.7 Selectivity

The HPLC analytical method is specific and robust under the specified conditions. No interference was detected. Under the specified operating conditions, the method was able to detect also the isomer 4-methyl-4-penten-2-one [3744-02-3].

## 11 Discussion

The analytical method described above is suitable for determining 4-methyl-3-penten-2-one in the workplace air in a concentration range from one tenth to twice the currently valid MAK value or OELV of 8.1 mg/m^3^. The method is suitable for monitoring compliance with the short-term concentration.

The experiments show that some of the adsorbed 4-methyl-3-penten-2-one is lost with the flow of the air sample at a relative humidity of 60% and above and a sampling period of 120 minutes. Under these conditions, the sampling period must be shortened and/or the flow rate must be reduced. Other options for adjusting the sampling conditions in case of high relative humidity are described in Section 10.3.

In general, all conditions must be adapted to the specific HPLC system that is used.
